# Age-associated Impairment of the Mucus Barrier Function is Associated with Profound Changes in Microbiota and Immunity

**DOI:** 10.1038/s41598-018-35228-3

**Published:** 2019-02-05

**Authors:** Bruno Sovran, Floor Hugenholtz, Marlies Elderman, Adriaan A. Van Beek, Katrine Graversen, Myrte Huijskes, Mark V. Boekschoten, Huub F. J. Savelkoul, Paul De Vos, Jan Dekker, Jerry M. Wells

**Affiliations:** 1 Top Institute Food and Nutrition, Wageningen, The Netherlands; 2 Host-Microbe Interactomics Group, Wageningen University and Research Center, Wageningen, The Netherlands; 3 Cell Biology and Immunology Group, Wageningen University and Research Center, Wageningen, The Netherlands; 4 University of Groningen, University Medical Center Groningen, Groningen, The Netherlands; 5 Laboratory of Microbiology, Wageningen University and Research Center, Wageningen, The Netherlands; 6 Division of Human Nutrition, Wageningen University and Research Center, Wageningen, The Netherlands

## Abstract

Aging significantly increases the vulnerability to gastrointestinal (GI) disorders but there are few studies investigating the key factors in aging that affect the GI tract. To address this knowledge gap, we used 10-week- and 19-month-old litter-mate mice to investigate microbiota and host gene expression changes in association with ageing. In aged mice the thickness of the colonic mucus layer was reduced about 6-fold relative to young mice, and more easily penetrable by luminal bacteria. This was linked to increased apoptosis of goblet cells in the upper part of the crypts. The barrier function of the small intestinal mucus was also compromised and the microbiota were frequently observed in contact with the villus epithelium. Antimicrobial Paneth cell factors Ang4 and lysozyme were expressed in significantly reduced amounts. These barrier defects were accompanied by major changes in the faecal microbiota and significantly decreased abundance of *Akkermansia muciniphila* which is strongly and negatively affected by old age in humans. Transcriptomics revealed age-associated decreases in the expression of immunity and other genes in intestinal mucosal tissue, including decreased T cell-specific transcripts and T cell signalling pathways. The physiological and immunological changes we observed in the intestine in old age, could have major consequences beyond the gut.

## Introduction

Ageing is an ill-defined process involving changes in various body systems, which converts a mature, fit person into an increasingly infirm one. With the passage of time, individuals show decreasing cell-protection mechanisms and detrimental physiological changes in metabolic processes and physiological functions of various tissues including the heart, brain, and skeletal muscles^1^. This leads to increased morbidity and mortality due to autoimmune diseases, cancer and infectious disease^2,3^, as well as a decline of mental health, well-being, and cognitive abilities^4,5^.

One of the most important effects of the ageing process is a significant decline of the efficacy of both the adaptive and innate immune systems, which has been described for several species^6,7^. Furthermore, one study on oral and parenteral vaccination in naturally ageing mice showed that age-associated decrease in antigen-specific immune responses occurs earlier in the mucosal immune system than in systemic immune system^8^.

Aging significantly increases the vulnerability to gastrointestinal (GI) disorders with approximately 40% of geriatric patients reporting at least one GI complaint during routine physical examination^9^. Despite the need to further understand age-associated factors that increase the susceptibility to GI dysfunction, there is a paucity of studies investigating the key factors in aging that affect the GI tract. To date, studies in rodents have demonstrated that aging alters intestinal smooth muscle contractility^10^, as well as the neural innervations of the GI tract musculature^11^. Several studies in rodents have also reported an increase in intestinal permeability to macromolecules with age^12,13^. Specifically, advancing age was shown to correlate with an enhanced transepithelial permeability of D-mannitol, indicating that there may be an age-associated decline in barrier function^14^. In humans, the decreased intestinal motility results in a slower intestinal transit that affects defecation and leads to constipation^15^. The elderly are at a higher risk for infections, especially severe infections, as well as for certain autoimmune diseases and cancer, and their immune responses to vaccination are diminished^16^. It is considered that aged humans exhibit a loss of naive T cells and a more restricted T cell repertoire^17^. Furthermore, aging results in decreased human CD8+ cytotoxic T lymphocyte responses, restricted B cell clonal diversity, failure to produce high-affinity Abs, and an increase in memory T cells^18,19^. It has been suggested that although certain dendritic cell (DC) populations are fully functional in ageing^20,21^, both foreign and self-antigens induce enhanced pro-inflammatory cytokines^22^. Very old individuals with a more balanced pro- and anti-inflammatory phenotype may be the most fortunate^23,24^. The association of inflammation in ageing has been termed ‘inflammageing’^25^.

Human microbiome analyses have revealed significant changes in the intestinal microflora specifically with an increase of *Bacteroides* ssp in the elderly (<65 years)^26,27^. However, other authors have concluded that the change in the microbiota was seen only in centenarians with increased inflammatory cytokine responses, but not in elderly with an average age 70 ± 3 years)^28^. In centenarians, the microbiota differs significantly from the adult-like pattern, by having a low diversity in terms of species composition. *Bacteroidetes* and *Firmicutes* still dominate the gut microbiota of extremely old people (representing over 93% of the total bacteria). However, in comparison to the younger adults, specific changes in the relative proportion of *Firmicutes* subgroups were observed, with a decrease in the contributing *Clostridium* cluster XIVa, an increase in Bacilli, and a rearrangement of the *Clostridium* cluster IV composition^28^. Moreover, the gut microbiota of centenarians is enriched in *Proteobacteria*, a group containing “pathobionts”, shown to cause harm in a compromised or susceptible host^29,30^.

For maintaining the mammalian intestinal homeostasis with the microbiota, a key element is to minimize and regulate contact between luminal microorganisms and the intestinal epithelial cell surface. In the small intestine, physical separation of bacteria and the epithelium is largely accomplished by secretion of mucus, antimicrobial proteins, and IgA into the lumen^31,32^. Intestinal mucus is primarily composed of the highly *O*-glycosylated mucin 2 (Muc2), which is secreted by goblet cells in the epithelium. In the mouse colon, less antimicrobial peptides are secreted, therefore, a thick stratified inner layer is needed to separate the commensal microbes from the epithelium^33^. Both mucus layers have essentially the same composition, suggesting the outer mucus layer arises from limited proteolytic cleavage and volumetric expansion of the inner layer. The density and stratified organization of the inner mucus layer is proposed to prevent penetration by bacteria thereby minimizing and regulating contact between bacteria and the epithelium^33^.

Muc2 is the major secreted intestinal mucin and its absence in Muc2^−/−^ mice leads to colitis, which starts in the distal colon and spreads to the proximal colon^34,35^. Colitis is associated with increased microbiota diversity and an early colonization with pathobionts such as *Bacteroides fragilis*^36^. Moreover, it has been shown that even decreased Muc2 production, as observed in Muc2^+/−^ mice perturbs intestinal homeostasis and microbiota composition. Decreased mucus production is also observed in other mouse models of colitis leading to increased epithelial contact with bacteria as observed in Muc2^+/−^ mice^37,38^.

Several studies have shown age-associated effects on various components of the intestinal barrier and immune system (9). A recent study in accelerated Aging *Ercc1*^*−/Δ7*^ mice showed that a decline in the mucus barrier occurs by 16 -weeks of age^39^. Knowledge of the impact of ageing on the GI tract mucus layer of naturally aged mice is incomplete and limited to reports of altered gastric mucus layer^40^ and colonic mucus in 38-week old rats^41^. Moreover, none of the above-mentioned studies in naturally aged rodents have deeply investigated the genome-wide effects of ageing in the physiology of the small and large intestine using transcriptomics combined with other techniques such as histology and microbiota profiling. Such knowledge might provide new insights into the dynamics of the interplay between the host and microbiota in elderly and have implications for future interventions, for example by manipulation of the microbiota. To address this knowledge gap, we took advantage of 10-week- and 19-month-old litter-mate mice, which provides an opportunity to identify microbiota and host gene expression changes in association with ageing. Although mice have a lifespan of about 28 months, we hypothesise that 19-month-old mouse will develop significant changes related to age in their intestinal physiology, which might lead to altered microbiota-host interactions and altered intestinal physiology. Microbiota composition was determined and transcriptomics data was obtained from colonic tissue. Furthermore, (immuno)-histology and FISH techniques were performed on mouse colonic tissue to obtain temporal data on morphological changes, mucus production, and compartmentalization of bacteria in the lumen.

## Results

### Aged mice have fewer viable goblet cells in the colon than young mice as well as longer villi and reduced expression of Paneth cell markers in the ileum

**Figure 1:**
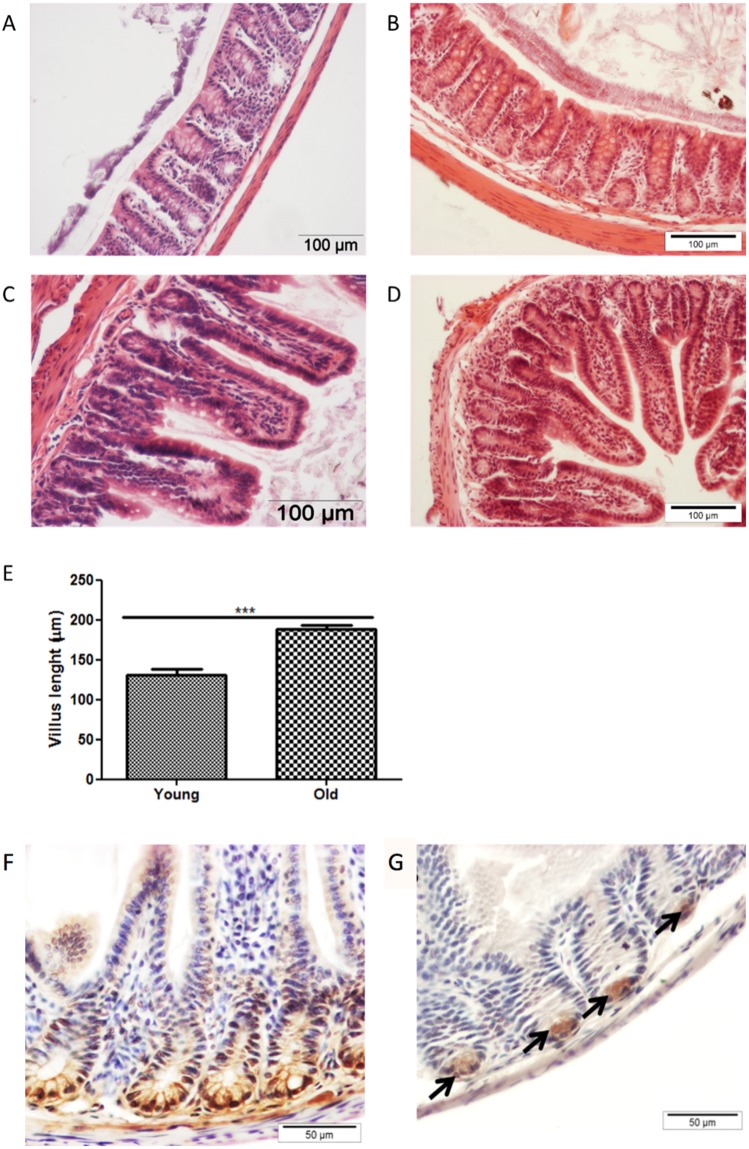
Representative images of H&E staining of colon (**A**) and ileum (**C**) of young (10 week-old) mice, and of colon (**B**) and ileum (**D**) of old (19 month-old) mice. Scale bar: 100 μm. Villus length measured on 10 well-oriented villi (5 mice per group). Pooled villus length measurement are presented in panel E. ***Indicates statistical difference at P < 0.001. Representative images of histochemical staining for lysozyme-P in ileal tissues from young (**F**) and old (**G**) mice. Scale bar: 50 μm.

**Figure 2:**
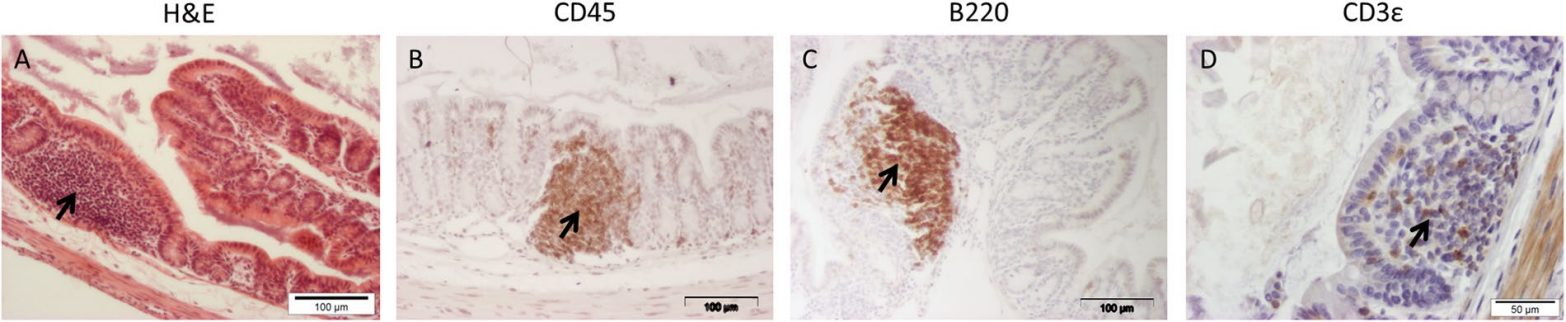
Representative pictures of Solitary Intestinal Lymphoid Tissue (SILT) stained with H&E (**A**), CD45 (**B**), B220 (**C**) and CD3ε (**D**) in old mice. Scale bar: 50 and 100 μm.

**Figure 3:**
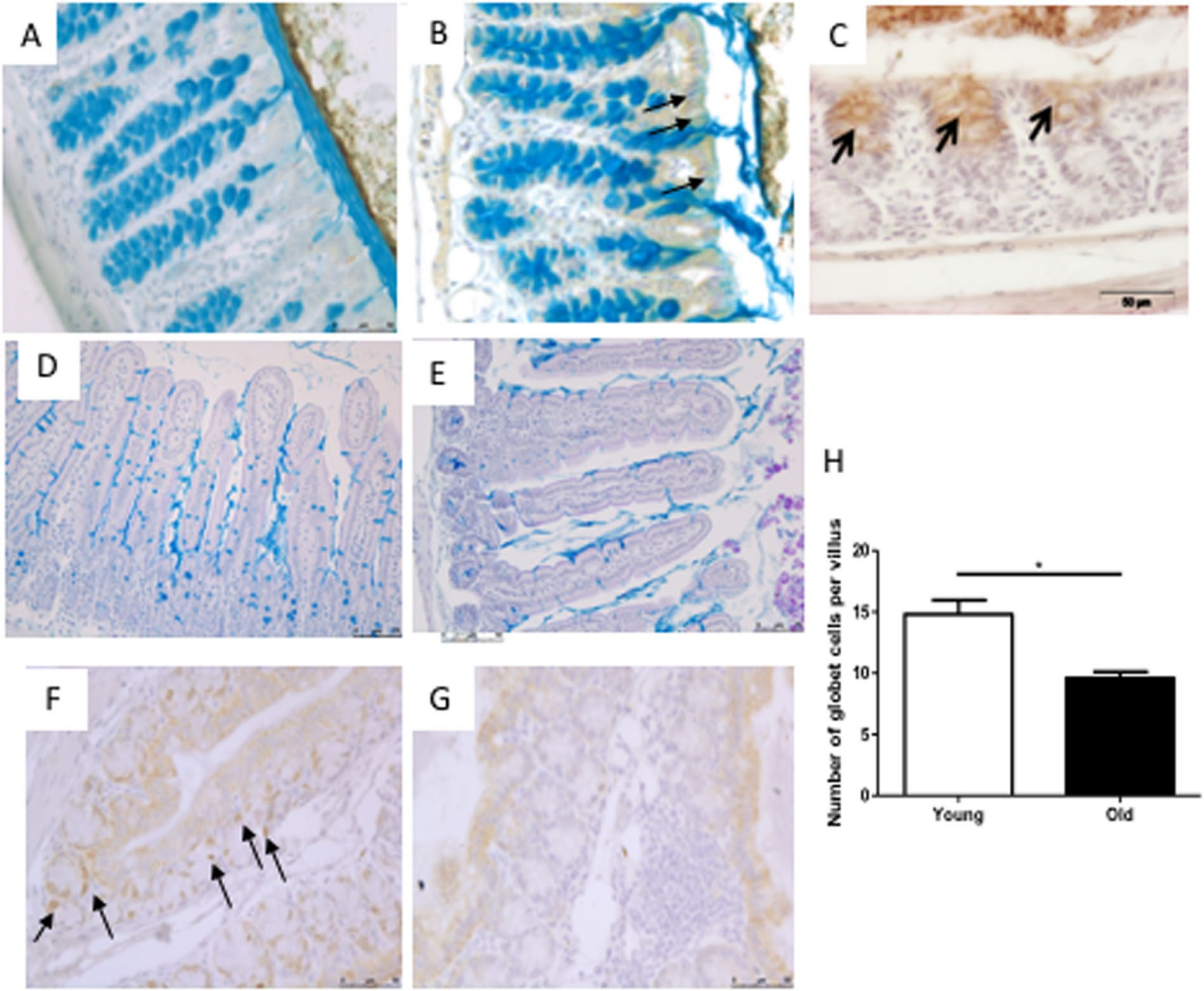
Colon sections of 10 week-old (**A**) and 19 month-old mice (**B**) stained with Alcian blue to reveal goblet cells and antibody to cleaved caspase 3 to reveal apoptotic cells. (**B** and detail in panel (C) A proportion of goblet cells in the in colonic crypts of 19-month-old mice but not in 10-week-old mice stained positive for cleaved caspase 3 (arrowed). Ileum sections of 10 week-old mice (**D**) and 19 month-old mice (**E**) stained with Alcian blue to reveal goblet cells. Significantly greater number of goblet cells per villus were observed in 10 week-old mice than 19 month-old mice (**H**). Immunostaining for Ki67 revealed more strongly positive proliferative cells in colon sections of the 10 week-old mice (black arrows) than 19 month-old mice (**F**,**G**).

There were no obvious histological differences in the H&E stained colon (Fig. 1A,B) and ileum (Fig. 1C,D) sections of 19-month-old and 10-week-old mice. However, in 19-month-old mice the villi were significantly (P < 0.001) longer (Fig. 1E) and there were fewer Paneth cells than in younger mice (Fig. 1F,G). Solitary intestinal lymphoid tissue (SILT) structures^42^ were more frequently observed in the colonic mucosa of 19-month-old mice than 10-week-old mice. However, SILT structures are known to develop postnatally at various locations throughout the colon so it is not possible to draw firm conclusions without use of a technique which examines the whole intestine. These lymphoid structures comprised mainly of CD45-positive haematopoietic cells and some CD3-positive T-cells (Fig. 2). Immunostaining for cleaved caspase 3 and mucus-containing goblet cells revealed increased apoptosis of cells in the upper part of the colonic crypts of 19-month-old mice than 10-week-old mice, but no obvious reduction in total goblet cell numbers in the colon (Fig. 3A,B). A proportion of goblet cells in the in colonic crypts of 19-month-old mice but not in 10-week-old mice stained positive for cleaved caspase 3 which may account for the reduced thickness of secreted mucus in old mice (Fig. 3C). No compensatory increase in epithelial cell proliferation (Ki67 staining) was observed to counter balance the apoptosis of goblet cells (Fig. 3G,F). These findings suggested that aged mice would produce less secreted mucus than young mice due to the presence of fewer vital goblet cells. The 19-month-old mice had significantly fewer goblet cells in the ileum than 10-week-old mice (Fig. 3D,E,H).

### The colonic mucus layer is thinner or absent in aged mice resulting in a failure to spatially compartmentalise the microbiota to the intestinal lumen

**Figure 4:**
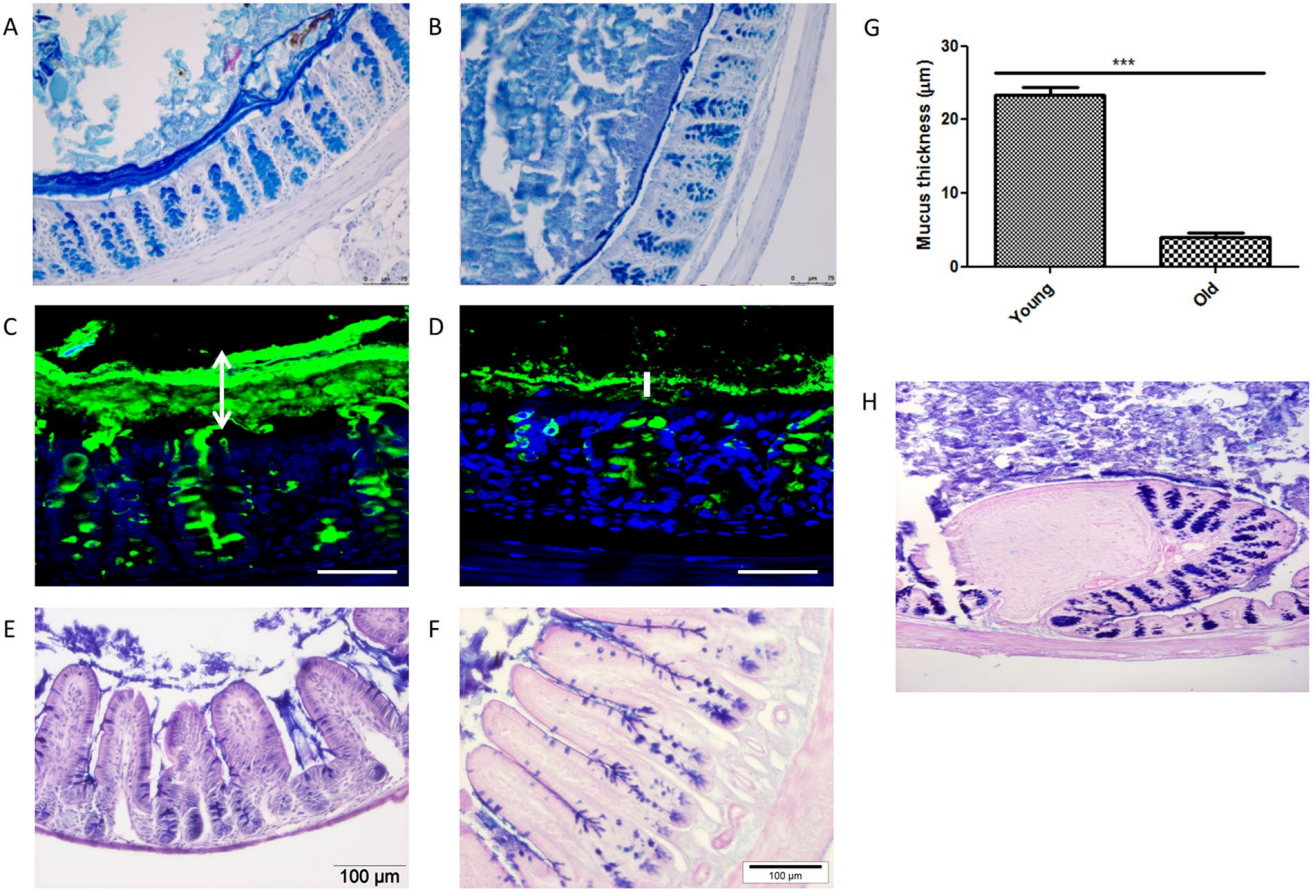
Representative pictures of PAS/Alcian Blue staining of colon (**A**) and ileum (**E**) of 2 month-old mice, and of colon (**B**) and ileum (**F**) of 19 month-old mice. Scale bars: 100 μm. Muc2 staining (in green; cell nuclei in blue) of colon of young mice (**C**) and old mice (**D**). Scale bars: 50 μm. Mucus thickness measured (10 measurements per section, 2 sections per animal) in 5 colonic tissues of young and old mice, respectively. Pooled mucus measurements are presented in panel G. ***Indicates statistical difference at P < 0.001. Representative pictures of PAS/Alcian Blue staining of colonic SILT (**H**).

Alcian blue-staining was used to identify acidic carbohydrates like Muc2, and Periodic Acid Schiff (PAS) for neutral carbohydrates, both of which occur on the Muc2 glycoprotein. Previous studies showed that the inner mucus layer of the proximal colon of young wild-type mice forms a stratified layer of Muc2 mucin, physically separating bacteria from the epithelium. The colonic mucus layer was absent or much thinner (1–5 µm) in 19-month-old mice than 10-week-old mice (Fig. 4A–G). Additionally, we observed that mucus layer was absent over the epithelium covering the SILT which was more abundant in the colon of old mice than young mice. This was due to the lack of mucus-secreted goblet cells in this area (Fig. 4H).

**Figure 5:**
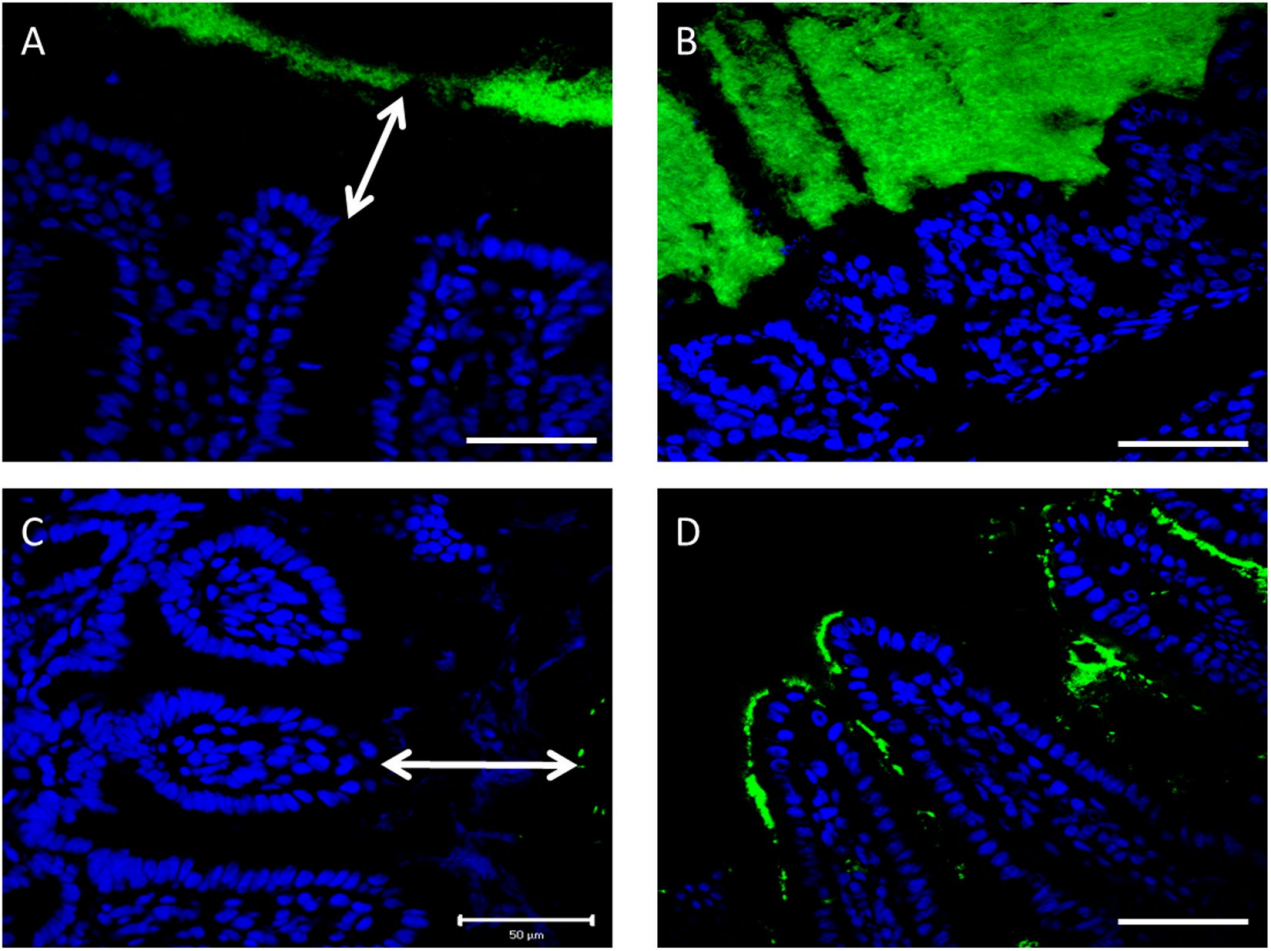
FISH analysis of sections of the colon (**A**) and ileum (**C**) of young mice using the general bacterial probe EUB338-Alexa Fluor 488 (green), and nuclear staining DRAQ5 (blue), and similar analysis of sections of the colon (**B**) and ileum (**D**) of old mice. Arrows indicate the distance between bacteria and epithelium. Scale bars: 50 μm.

The effect of ageing on the spatial compartmentalisation of bacteria in both small intestine and colon was investigated by fluorescent *in situ* hybridization (FISH). In aged mice the thinner mucus layer was associated with increased bacterial penetrability and contact with the epithelium. For example, in small intestine and colon of young mice we observed a clear “gap” of about 50 μm in between the microbiota and the epithelium (Fig. 5A,C). This “gap” corresponded to the thick mucus layer between the surface of the epithelium and the luminal content (Fig. 4). However, at many locations in the small intestine and colon of 19-month-old mice the microbiota was frequently observed in direct contact with epithelial surfaces, due to lacunas in the mucus layer, which was never observed in young mice (Fig. 5B,D).

### Alterations in innate and adaptive immune responses are observed in the large and small intestine of ageing mice

In the large intestine, a transcriptomics approach was used to gain more insight into the potential pathways and mechanisms that might be modulated by the dysfunctional barrier functions observed in aged mice (above). Applying the following criteria, fold-changes >1.2, p-value < 0.05 and signal intensity >20 in at least one of the arrays, we found 1503 differentially expressed genes (759 up-regulated and 744 down-regulated) in old mice versus young mice (data not shown).

**Figure 6:**
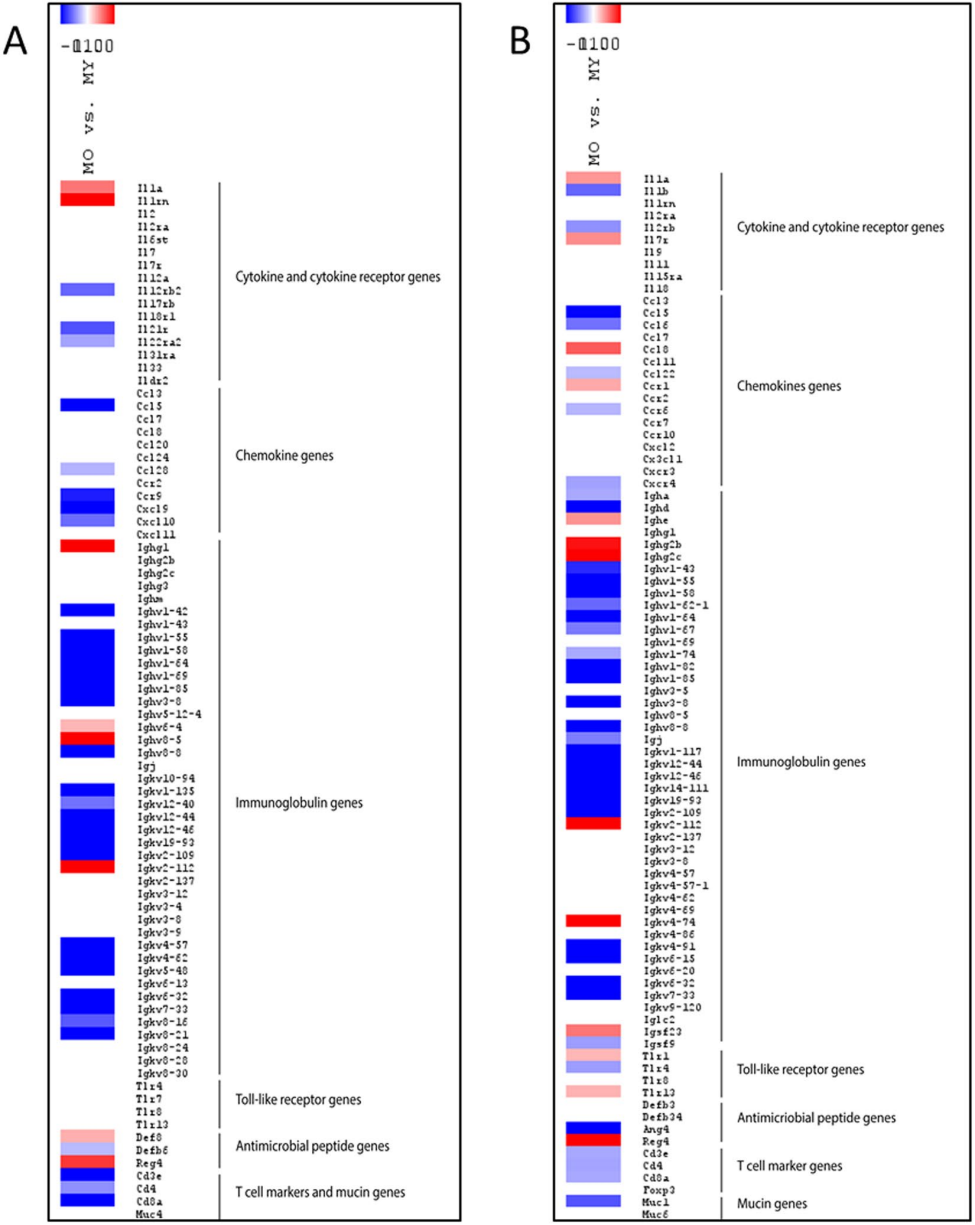
Heatmaps of a selected panel of immunity-related genes expressed in proximal colon (**A**) and ileum (**B**). Genes differentially expressed in old (O) versus young (Y) mice which are upregulated are shown in red and genes that are down-regulated are shown in blue. Scale: Log2(Fold Change) = −1 (blue) <0 (white) <1 (red). Genes in white are not differentially expressed between old and young mice and have fold changes between −1 and +1. The intensity of the red and blue colours is proportional to change in expression. Ighv and Igkv gene annotations are variable regions of the kappa and heavy immunoglobulin chains; differences in expression of these genes is interpreted as indicating altered antibody expression by plasma cells in the mucosa of old vs young mice.

A Heatmap was generated for immunity-related genes, including those that encode Pattern Recognition Receptors (PRRs), cytokines, chemokines, immunoglobulins, antimicrobial (poly)peptides, T cell markers (CD3ε), and T-helper (CD4 or CD8) and Tregs (Foxp3) subsets that were differentially expressed in the proximal colon of 19-month-old mice (Fig. 6A). The down-regulated *Cd3ε*, *Cd4*, and *Cd8* suggest a decreased abundance of T cells. The strong down-regulation of chemokine genes and immunoglobulin expression suggest a decrease in immune responsiveness and B cell activity.

In the ileum 930 genes were found differentially expressed (428 up-regulated and 502 down-regulated) in old versus young mice (data not shown). The Gene Ontology Biological Processes annotations of the differentially expressed genes showed that predominantly processes related to adaptive immunity were strongly down-regulated in the small intestine. The most down-regulated pathways were CTLA4 Signalling in Cytotoxic T Lymphocytes, T Cell Receptor Signalling, Natural Killer Cell Signalling, Role of NFAT in Regulation of the Immune Response, CD28 Signalling in T Helper Cells (Supplementary Fig. 1). The antimicrobial protein angiogenin 4 (Ang4) that is produced by Paneth cells was strongly down-regulated in the ileum of old mice.

A similar Heatmap as for colon was generated for immunity-related genes including those that encode Pattern Recognition Receptors (TLRs etc.), cytokines, chemokines, immunoglogulins, antimicrobial (poly)peptides, T cell markers (CD3ε) and T-helper (CD4 or CD8) and Tregs (Foxp3) subsets that were differentially expressed in the ileum of 19-month-old males (Fig. 6B). As in colon, genes related to innate and adaptive immunity were strongly down-regulated in old mice compared to young mice.

### Ageing is associated with altered intestinal microbiota

**Figure 7:**
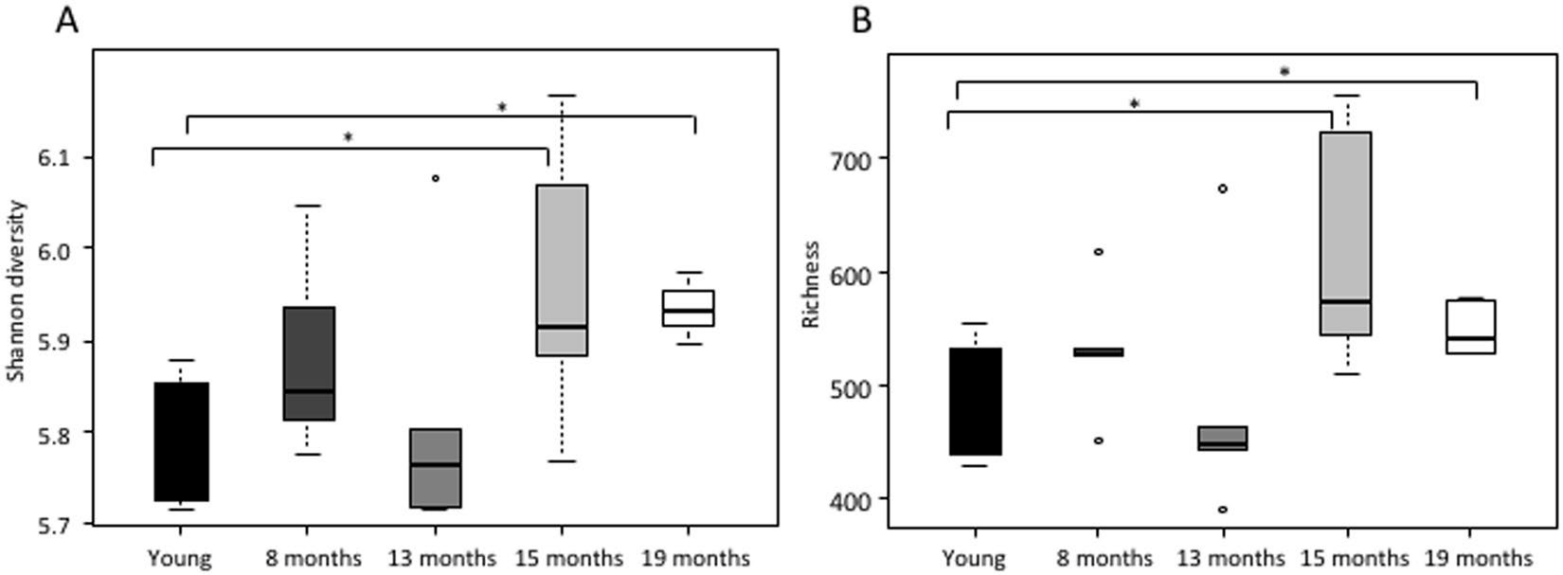
Box-and-whisker plot showing the bacterial diversity (Shannon index) of faecal samples from 10-week-old, 8-month-old, 13-month-old, 15-month-old, and 19-month-old mice (**A**). Box-and-whiskers-plot showing the richness of microbiota in colon of young, 8 months, 13 months, 15 months, and 19 months of age (**B**). Statistically significant differences among groups and time points were indicated (*P < 0.05).

**Figure 8:**
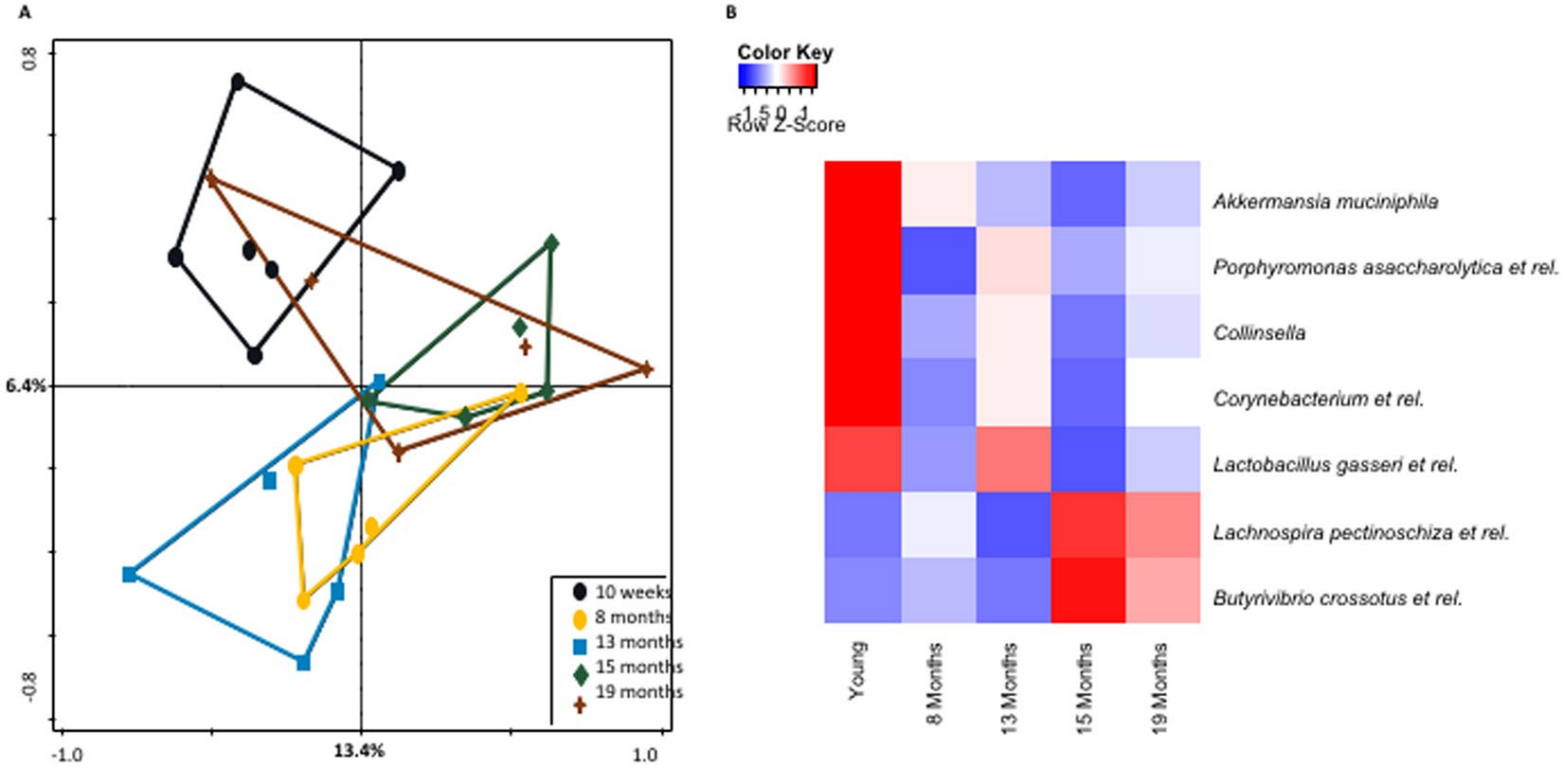
(**A**) Redundancy Analysis (RDA) plot representing microbial ecology of young (10 week-old) mice (black cluster), 8 months (yellow cluster), 13 months (blue cluster), 15 months (green cluster) and 19 months (brown cluster) in the faeces. The different ages were used as explanatory variables for the RDA, where the 10-week-old and 13-month-old variables had significantly different microbial compositions (Monte Carlo permutation test; P < 0.05). (**B**) Heatmap showing the abundance fold-change of bacteria with age. In red, bacteria which are significantly more abundant in faeces and in blue the bacteria that are less abundant. Scale: Log2(Fold Change) = −1 (blue) <0 (white) <1 (red). The intensity of the colour is proportional to change in bacterial presence.

To investigate the impact of ageing on the colonization pattern of the colon, 16S rRNA microbiota profiles of faeces from 10-week-, 8-month-, 13-month-, 15-month-, and 19-month-old C57BL/6 mice were determined using the MITChip microarray^43^. The faecal content of 15 - and 19 months-old mice displayed both significantly increased richness and α-diversity compared to young mice (P = 0.036 and P = 0.0014, respectively), (Fig. 7). The intermediate ages (8 and 13 months) displayed similar diversity and richness as the young mice, although diversity was statistically higher at 8 months compared to 10-week- and 13-month-old mice (Fig. 7). Nevertheless, redundancy analysis (RDA) clearly established that at 8, 13, 15, and 19 months of age the microbiota composition was clearly distinct compared to 10 week-old mice, with the exception of one mouse in the 19-month-old group, causing a partial overlap of the microbiota cluster with 8-month-old mice (Fig. 8). *Akkermansia muciniphila*, *Porphyromonas asaccharolytica et rel*., *Collinsella*, *Corynebacterium et rel*., and *Lactobacillus gasseri et rel*., were statistically less abundant (P < 0.05 with correction for multiple testing) in old mice compared to young ones (Fig. 8). *Lachnospira pectinoschiza et rel*., and *Butyrivibrio crossotus et rel*., were significantly more abundant in old mice (P < 0.05 with correction for multiple testing), (Fig. 8).

### Abundance of specific bacteria correlates with expression of genes in ageing mice

**Figure 9:**
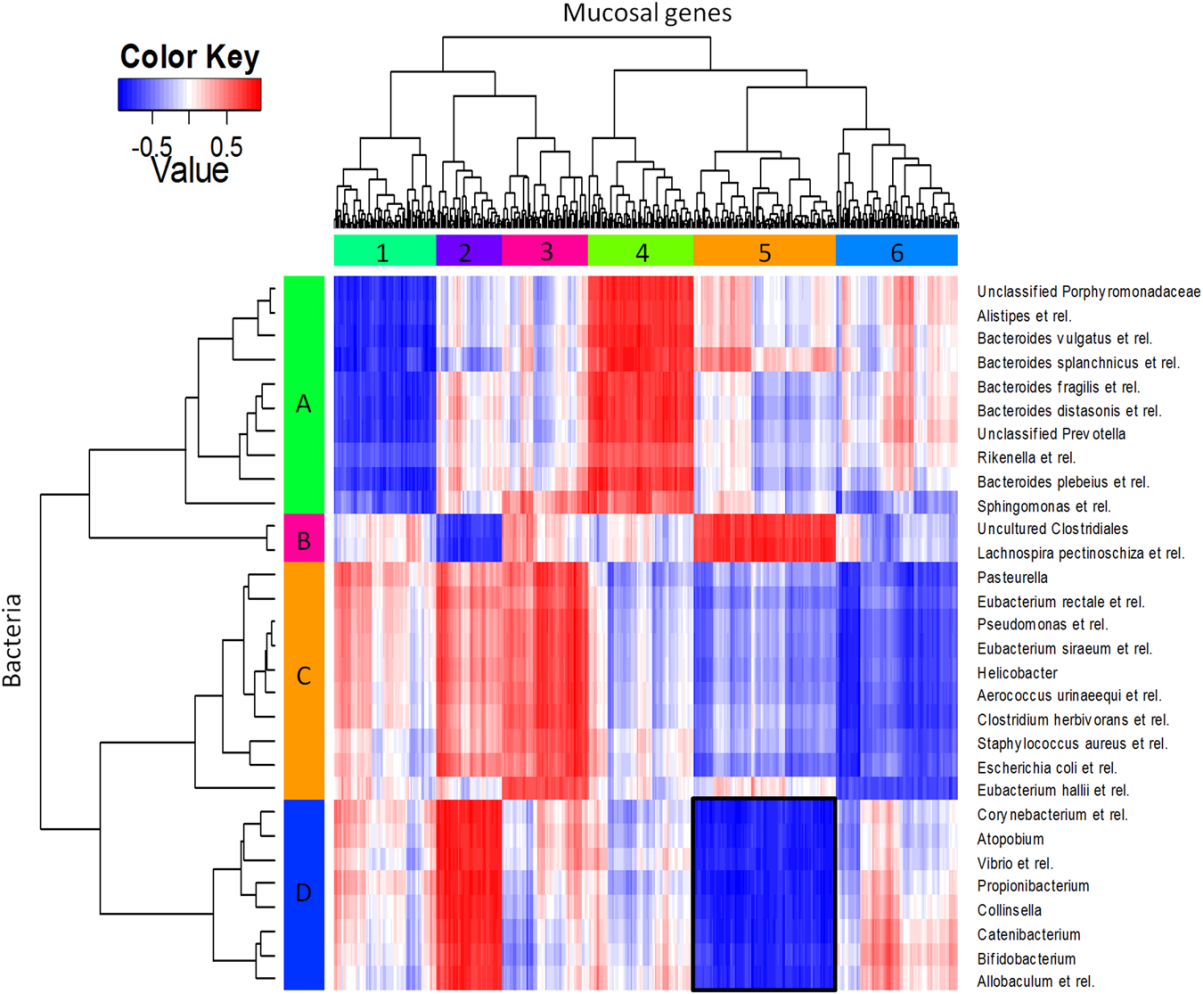
Heatmap of the correlation coefficients between faecal bacterial abundance (in rows) and mucosal gene expression levels (in columns) in 19-month-old mice. The integration of datasets was done per individual mice (5 mice per group) and gives direct correlation between gene expression and microbiota composition over these samples. In deep red, the cluster of genes that most positively correlated with one group of bacteria. In deep blue, the cluster of genes that most negatively correlated with a respective group of bacteria. The intensity of the colour is proportional to to the value of the correlation coefficient (colour scale in top left panel). Six main gene clusters (1–6) and 4 main bacterial clusters (**A**–**D**) were identified. Overrepresented GO-terms in the respective gene clusters are listed in Supplementary Table 1.

To identify bacteria that are correlated with gene expression changes in the colon of aged mice, we performed a multivariate integration and correlation analysis on the data from 19-month-old mice. Microbiota and transcriptomics data were pooled for individual mice to investigate direct correlation between gene expression and microbiota composition over these samples. Three bacterial clusters (A, B and D) strongly correlated either positively (red) or negatively (blue) with 4 clusters of genes (1, 2, 4 and 5; about 100 genes per cluster) (Fig. 9). The correlations between bacteria and cluster C were not included because they were relatively much weaker. The individual genes in the respective gene clusters are listed in Supplementary, Table 1. Strongest correlations were between microbiota members (cluster D on y axis, Fig. 9) and specific changes in mucosal gene expression (cluster 5 on x-axis), shown in Fig. 9. The bacteria of cluster D had higher relative abundances in the mice of 15- and 19-month-old mice compared to young mice and displayed a negative correlation with immune response genes involved in apoptosis that were differentially expressed in our transcriptomics data from 19-month vs 10-week old mice (Supplementary Data, Table 1, cluster 5 genes in bold). A positive correlation was found between bacteria in cluster D and genes listed in cluster 2, however transcriptomics data shown no significant differences between the relative expression of these genes in old and young mice.

## Discussion

In this study, we showed that 19-month-old male mice have absent or greatly reduced thickness of the colon mucus layer compared to 10-week-old mice (Figs 4 and 5). A decrease of about 5 μm in the thickness of colonic mucus was reported previously in 38 week-old rats compared to animals aged 4 weeks or 8 weeks^41^, but this reduction was much smaller than we observed in very old mice (19 months). The reduction of mucus secretion in the colon of 19-month-old mice is likely to be due to the striking increase in apoptotic goblet cells, in the upper part of the colonic crypts (Fig. 2). Despite the impairment of the mucus barrier in old mice and chronic exposure of the epithelial surface to luminal bacteria (Fig. 4), there was no histological evidence of colitis (Fig. 1). However, in *Muc2*^−/−^ mice which lack a secreted mucus layer spontaneous colitis occurs around 5 to 6 weeks of age. Taken together these observations suggest that production of a small amount of intestinal mucus is sufficient to avoid colitis development, even when the microbiota is not compartmentalised to the lumen. This may be due to proposed immune regulatory effects of mucin itself and, or its crucial role in lubrication of faecal passage and the avoidance of mechanical stress on the gut wall. Another factor influencing the risk of colitis may be the age-related down-regulation of host inflammatory immune responses as evidenced by the transcriptomics study on colon and ileal tissue. Transcription of genes related to innate and adaptive immunity such as immunoglobulins (in particular IgA) were strongly down-regulated in both the colon and ileum of old mice (Fig. 6A,B).

In the colon SILT structures were observed more frequently in 19-month-old mice than 10-week old mice. SILT are known to develop early after birth and are dependent on exposure to intestinal microbiota^44^. Thus, increased contact with intestinal microbiota induced by ageing and mucus shrinkage might explain the more frequent occurrence of colonic SILT in old mice. The SILT were mainly composed of B-cells confirming the results of a previously published study^42^.

The mucus barrier also appeared to be compromised in the ileum of 19-month-old mice and bacteria were frequently seen in contact with the epithelium (Fig. 4). As found in the colon, the transcriptome data revealed a down-regulation of innate and adaptive immune genes including immunoglobulins (in particular IgA), TLR4, T cell (CD3ε) and T-helper (CD4 or CD8) markers and the antimicrobial factor Ang4 were strongly down-regulated in both the colon and ileum of old mice. Histology showed that the Paneth cell marker lysozyme was also strongly down-regulated in the ileum. Secretory IgA and antimicrobial factors play a key role in regulating contact between the epithelium and potentially harmful antigens and microbes^31,32^ and may explain our observation that the microbiota were frequently seen in contact with the villus epithelium (Fig. 5). Decreased mucus production in the ileum of old mice may also be a contributing factor. Although we did not measure any significant age-associated changes in Muc2 transcription decreased production in old mice could be due to altered post-translational processes. The only mucin gene differentially expressed in the ileum of old mice was the membrane tethered mucin *Muc1*, which was down-regulated. Interestingly the thickness of the secreted colonic mucus is decreased in *Muc1*^−/−^ mice, despite there not being a decrease in *Muc2* transcription compared to wild-type mice^45^.

Reg4 was of the few gene transcripts expressed in relatively higher amounts in the colon and ileum of old mice. The function of this gene is not well understood but is known to be up-regulated in cancer-initiating cells and was recently shown to be a specific marker for enteroendocrine cells in the intestine.

In previous studies, analyses of human microbiome in the elderly (>65 years) have revealed significant changes in the intestinal microflora specifically with an increase of *Bacteroides* ssp and distinct abundance patterns of *Clostridium* groups^26,27^. However, others have shown that the change in the microbiota was seen only in centenarians with increased inflammatory cytokine responses, but not in the general elderly population (average age 70 ± 3 years)^28^. To identify bacteria that might be correlated with changes in colon gene expression we used the linear multivariate method partial least squares (PLS) method^46^ for each time point, as previously described (Lange *et al*., in revision). We found that uncultured *Clostridiales* and *Lachnospira pectinoschiza et rel*. had higher relative abundances in the old mice of 15 and 19 months of age compared to young mice and displayed a positive correlation with immune response genes. A positive correlation was also found with stress response genes involved in apoptosis and cell proliferation, as well as immune genes. *Akkermancia municiphilia* was strongly decreased with ageing which has been observed in humans. *Akkermansia muciniphila* is a Gram negative bacterium, which in mice is the only species belonging to the phylum *Verrucomicrobia*^47^. Oral administration of obese and type 2 diabetic mice with viable *A. muciniphila* reversed high-fat diet-induced metabolic disorders, including fat-mass gain, metabolic endotoxemia, adipose tissue inflammation, and insulin resistance^48^. Furthermore, it has been shown that extracellular vesicles produced by *A. muciniphila* decrease expression of IL-6 in colonic epithelial cells and when orally administered to mice protect against DSS-induced colitis^49^.

In summary we showed that old mice have an impaired mucus barrier in the colon and ileum accompanied by major changes in the faecal microbiota composition and expression of immunity and other genes in intestinal mucosal tissue, leading to decreased T cell-specific transcripts and T cell signalling pathways. Given the important role of gut homeostasis and the microbiota in the aetiology of many diseases, the physiological and immunological changes we observed could have major consequences beyond the gut. An age-associated decline in antigen specific immune responses to oral vaccination has been reported to occur earlier than in the systemic immune compartment. Thus, the decline in gut barrier function may even be the trigger for the low-grade chronic inflammation or “inflammageing” typical of old age. In the future, prospective studies are needed to determine the sequence of events leading to the profound changes we observed in the intestinal mucosa of old mice as this will be important for future strategies aimed reducing the risk of infectious disease, colon cancer and potentially other diseases.

## Methods

### Animals and ethical approval

C57BL/6 mice (Harlan Laboratories, USA) were housed in a specific pathogen-free environment with ad libitum access to D12450B diet (10% fat) (Research Diets Services BV, Wijk bij Duurstede, the Netherlands), and acidified tap water in a 12-hour light/dark cycle. All animal experimental protocols were approved by the UMCG Animal Ethics Committee (Groningen, The Netherlands), and carried out in accordance with the approved guidelines.

### Experimental set up

Groups of 8-week-old males (n = 5) were housed in individual ventilated cages, and sacrificed at 2 months and 19 months of age. Ileal and colonic tissues were fixed in Carnoy’s fixative and embedded in paraffin as previously described^33^. Additionally, segments of ileum and colon were frozen in liquid nitrogen and stored at −80 °C for RNA and protein assays. Faecal samples were collected at 2, 8, 13, 15, and 19 months (sacrifice) and stored at −80 °C.

### Histology

Paraffin sections (5 µm) of ileum and colon were attached to poly-L-lysine-coated glass slides (Thermo scientific, Germany). After overnight incubation at 37 °C, slides were de-waxed and hydrated step-wise using 100% xylene followed by several solutions of distilled water containing decreasing amounts of ethanol. Sections were stained with hematoxylin and eosin (H&E) and PAS/Alcian blue^50^. Mucus layer thickness was measured (10 measurements per section/2 sections per animal/5 animals per condition) using Image J software (NIH, Maryland, USA).

### Immunohistochemistry

The slides were deparaffinised and antigen retrieval was performed by heating the sections for 20 min in 0.01 M sodium citrate (pH 6.0) at 100 °C. Sections were washed for 3 h with 3 changes of Tris-Buffered Saline (TBS). Non-specific binding was reduced using 10% (v/v) goat serum (Invitrogen, Life technologies Ltd, Paisley, UK) in TBS for 30 min at room temperature. T cells CD3 marker was detected by incubating the sections with anti-CD3 antibody (Invitrogen, Life technologies Ltd, Paisley, UK) diluted 1:100 in Tris-Buffered Saline (TBS), overnight at 4 °C. Leukocytes were detected by incubating the sections with anti-CD45 antibody diluted 1:100 in TBS, overnight at 4 °C. Paneth cells were identified staining for the lysozyme expression, detected by incubating the sections with anti-lysozyme antibody (Invitrogen) diluted 1:100 in TBS, overnight at 4 °C. Cell proliferation marker Ki67 was detected by incubating the sections with anti-Ki67 antibody (Abcam, Cambridge Science Park, Cambridge, UK) diluted 1:200 in TBS, 90 min at room temperature. Apoptotic cells were identified by staining for cleaved Caspase-3 expression using an anti-Caspase-3 antibody (Abcam) diluted 1:200 in TBS, overnight at 4 °C. Muc2 was detected by staining for the sections with anti-Muc2 antibody (kindly gifted by Dr. Gunnar Hansson) diluted 1:500 in TBS, and goat-anti-rabbit Alexa 488 conjugated antibody (1:1000) (Molecular Probes, Life Technologies Ltd, Paisley, UK) in TBS.

### Detection of bacteria using fluorescence *in situ* hybridization (FISH)

The slides were deparaffinised with xylene and rehydrated in a series of ethanol solutions to 100% ethanol. The tissue sections were incubated with the universal bacterial probe EUB338 (5′-GCTGCCTCCCGTAGGAGT-3′) (Isogen Bioscience BV, De Meern, the Netherlands) conjugated to Alexa Fluor488. A ‘non-sense’ probe (5′-CGACGGAGGGCATCCTCA-3′) conjugated to Cy3, was used as a negative control. Tissue sections were incubated overnight with 0.5 μg of probe in 50 μL of hybridization solution (20 mmol/L Tris-HCl (pH 7.4), 0.9 mol/L NaCl, 0.1% (w/v) SDS) at 50 °C in a humid environment using a coverslip to prevent drying of the sample. The sections were washed with (20 mmol/L Tris-HCl (pH 7.4), 0.9 mol/L NaCl) at 50 °C for 20 min and then washed 2 times in PBS for 10 min in the dark and incubated with DRAQ5 (Invitrogen) (1:1000) for 1 h at 4 °C to stain nuclei. Sections were washed 2 times in PBS for 10 min, mounted in fluoromount G (SouthernBiotec, Alabama, USA) and stored at 4 °C.

### Transcriptome analysis

Quantity and quality of colonic and ileal RNA (5 arrays of individual mice per group) was assessed using spectrophotometry (ND-1000, NanoDrop Technologies, Wilmington, NC, USA), and Bionanalyzer 2100 (Agilent, Santa Clara, CA, USA), respectively. RNA was only used to generate cDNA and perform microarray hybridisation when there was no evidence of RNA degradation (RNA Integrity Number >8). 100 ng of total RNA was labelled using the Ambion WT Expression kit (Life Technologies Ltd, Paisley, UK) together with the Affymetrix GeneChip WT Terminal Labelling kit (Affymetrix, Santa Clara, CA). Labelled samples were hybridised to Affymetrix GeneChip Mouse Gene 1.1 ST arrays. Hybridisation, washing, and scanning of the array plates were performed on an Affymetrix GeneTitan Instrument, according to the manufacturer’s recommendations.

Quality control (QC) of the datasets obtained from the scanned Affymetrix arrays was performed using Bioconductor^51^ packages integrated in an on-line pipeline^52^. Probe sets were redefined according to Dai *et al*.^53^ utilising current genome information. In this study, probes were reorganised based on the Entrez Gene database (remapped CDF v14.1.1). Normalised expression estimates were obtained from the raw intensity values using the Robust Multiarray Analysis (RMA) pre-processing algorithm available in the Bioconductor library affyPLM using default settings^54^.

Differentially expressed probe sets were identified using linear models, applying moderated t-statistics that implemented empirical Bayes regularization of standard errors^55^. A Bayesian hierarchical model was used to define an intensity-based moderated T-statistic (IBMT), which takes into account the degree of independence of variances relative to the degree of identity and the relationship between variance and signal intensity^56^. Only probe sets with a fold-change (FC) of at least 1.2 (up/down) and p value < 0.05 were considered to be significantly different. Biological interaction networks among regulated genes activated in response to ageing were identified using Ingenuity Pathways Analysis (IPA) (Ingenuity System). IPA utilizes a large expert-curated repository of molecule interactions, regulatory events, gene-to-phenotype associations, and chemical knowledge, mainly obtained from peer-reviewed scientific publications, that provides the building blocks for network construction. IPA annotations follow the GO annotation principle, but are based on a knowledge base of >1,000,000 protein-protein interactions. The IPA output signalling pathways with statistical assessment of the significance of their representation based on Fisher’s Exact Test. Our IPA analyses compared differentially regulated genes in the ileum and colon of old males compared to young mice. The input was all differentially regulated genes (p value < 0.05, FC > 1.2 and intensity >20) of ileum and colon.

### Bacterial DNA extraction and microbiota profiling

The DNA from ileal content and faeces was extracted using the repeated bead-beating-plus column method^57^. Microbiota composition was analysed by Mouse Intestinal Tract Chip (MITChip), a diagnostic 16S rRNA array that consists of 3,580 unique probes especially designed to profile mouse intestine microbiota^43^. 16S rRNA gene amplification, *in vitro* transcription and labelling, and hybridization were carried out as described previously^58^. The data was normalized and analysed using a set of R-based scripts in combination with a custom-designed relational database, which operates under the MySQL database management system. For the microbial profiling the Robust Probabilistic Averaging (RPA) signal intensities of 2667 specific probes for the 94 genus-level bacterial groups detected on the MITChip were used for the relative abundant measurements^59^. Diversity calculations were performed using a microbiome R-script package (https://github.com/microbiome), Shannon indices were plotted in a Box-and-whisker plot and statistical difference were tested by paired t-test between time-points. Wilcoxon signed-rank test was applied on the 94 genus-level bacterial groups to determine significant differences of individual bacterial groups between the 10-week-old and 19-month-old mice (Fig. 8B); when corrected for multiple testing all p values were higher than 0.05. Multivariate statistics, redundancy analysis (RDA), and Principal Response Curves (PRC), were performed in Canoco 5.0, and visualized in triplots or a PRC plot, and variables were tested for their significance in explaining the variation by Monte Carlo permutation test^60^.

### Multivariate integration and correlation analysis

To get insight into the interactions between changes in gene expression and microbiota composition, the datasets per time point were combined using the linear multivariate method partial least squares (PLS)^46^, as described before (Lange *et al*., in revision). By this integration, the two datasets were integrated per individual mouse. Both datasets were log2 transformed before analysis and the canonical correlation framework of PLS was used^61^. The correlation matrices were visualized in clustered image maps^62^. Analyses were performed in R using the library mixOmics^63^.

## Electronic supplementary material

Supplementary Information
