# Supplementary material for: Age-associated Impairment of the Mucus Barrier Function is Associated with Profound Changes in Microbiota and Immunity
Source: Sci Rep. 2019 Feb 5;9:1437. doi: 10.1038/s41598-018-35228-3 (PMC6363726; doi:10.1038/s41598-018-35228-3)
Supplement: Supplementary file 1 — Supplementary Information [file 41598_2018_35228_MOESM1_ESM.docx]

**Supplementary Information**

**Age-associated Impairment of the Mucus Barrier Function is Associated with Profound Changes in Microbiota and Immunity**

Bruno Sovran^1,3^, Floor Hugenholtz^5^, Marlies Elderman^1,4^, Adriaan A. Van Beek^1,3^, Katrine Graversen^2^, Myrte Huijskes^2^, Mark V. Boekschoten^1,6^, Huub F.J. Savelkoul^1,3^, Paul De Vos^1,4^, Jan Dekker^1,2^ and Jerry M. Wells^1,2*^

^1^Top Institute Food and Nutrition, Wageningen, The Netherlands; ^2^Host-Microbe Interactomics Group, Wageningen University and Research Center, Wageningen, The Netherlands; ^3^Cell Biology and Immunology Group, Wageningen University and Research Center, Wageningen, The Netherlands; ^4^University of Groningen, University Medical Center, Groningen The Netherlands; ^5^Laboratory of Microbiology, Wageningen University and Research Center, the Netherlands; ^6^Division of Human Nutrition, Wageningen University and Research Center, Wageningen, the Netherlands

* Corresponding author:

Prof. Jerry M. Wells,

P.O. Box 338, 6700 AH, Wageningen, The Netherlands

[jerry.wells@wur.nl](mailto:jerry.wells@wur.nl)

Tel: +31 (0)317 484509

Fax: +31 (0)317 483962


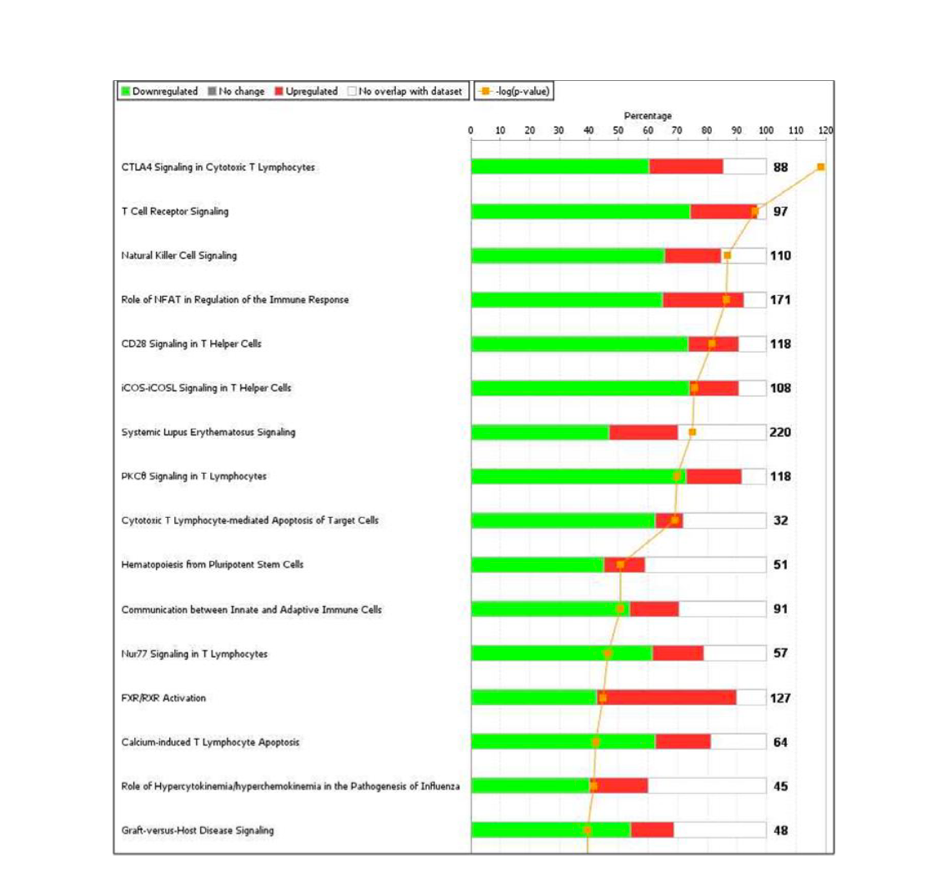


**Supplementary figure 1:** *Top 15 pathways significantly (-log(p value)>5) regulated in ileum of 19-month-old mice compared to young mice (10 weeks). In red the percentage of genes up-regulated, and in green the percentage of genes downregulated in each pathway.*

**Cluster 5 genes**


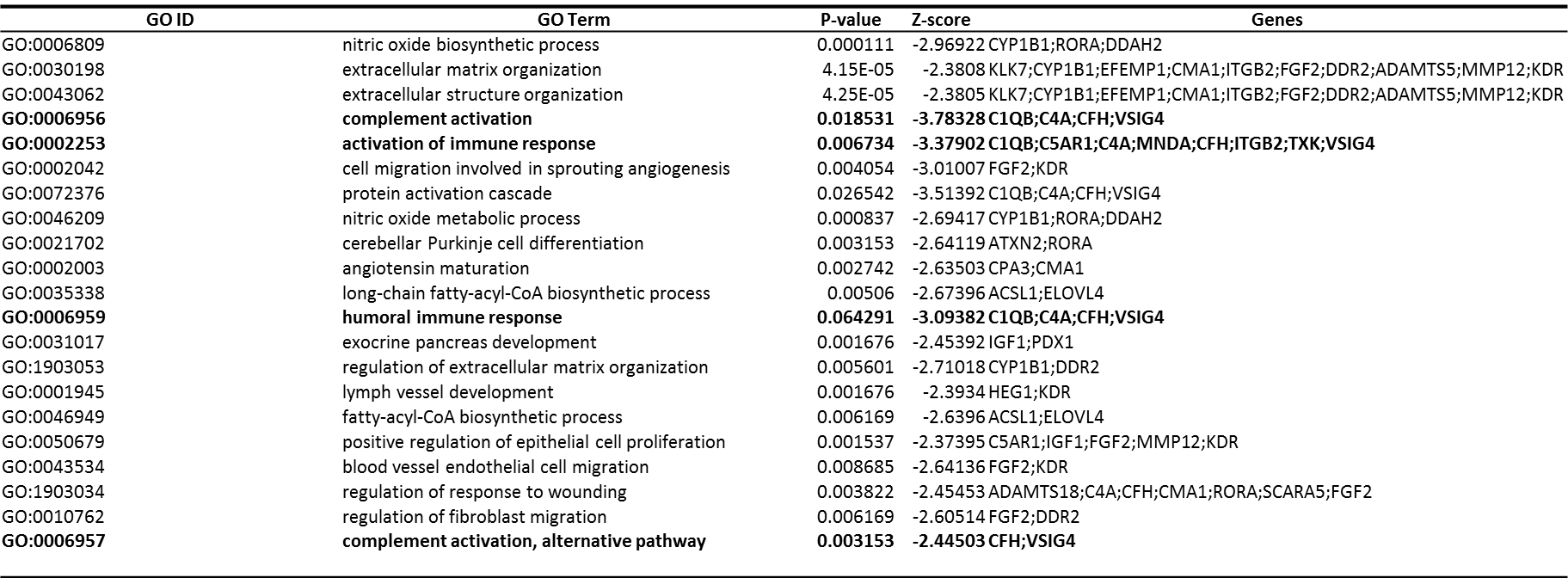


**Cluster 2 genes**


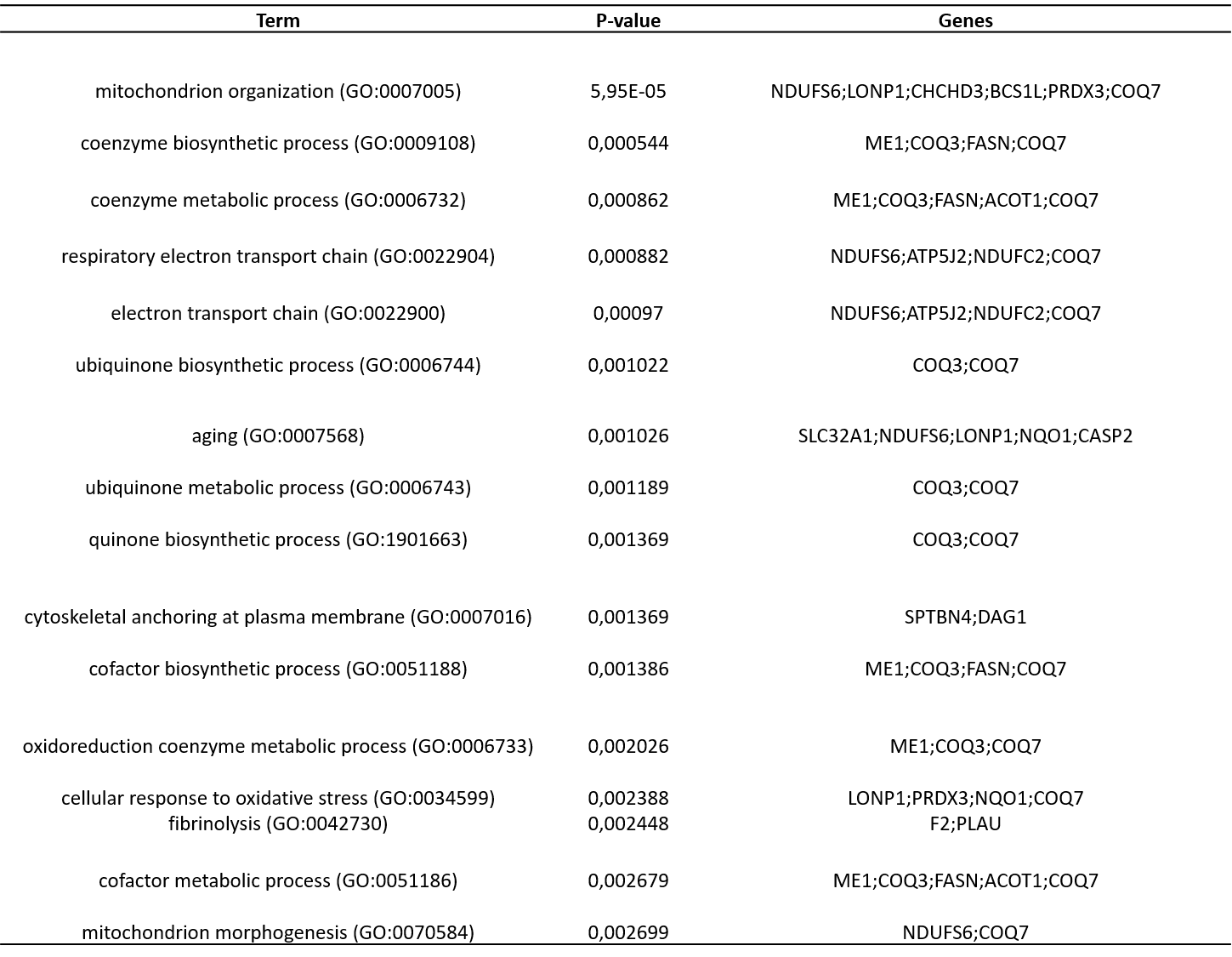


**Supplementary Table 1: (Above)** *Top 20 Gene ontology terms from the gene cluster 5 (Figure 10) negatively correlated with microbiota. In bold are depicted the genes (and associated GO terms) related to immunity***. (Below)** *Top Gene ontology terms from the gene cluster 2 (Figure 10) which are positively correlated with microbiota.*

*Cluster 1*

*
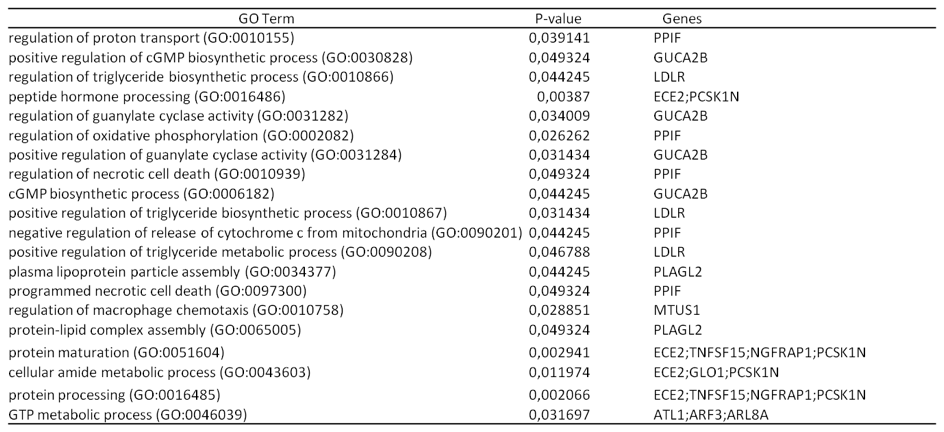
*

*Cluster 4*

*
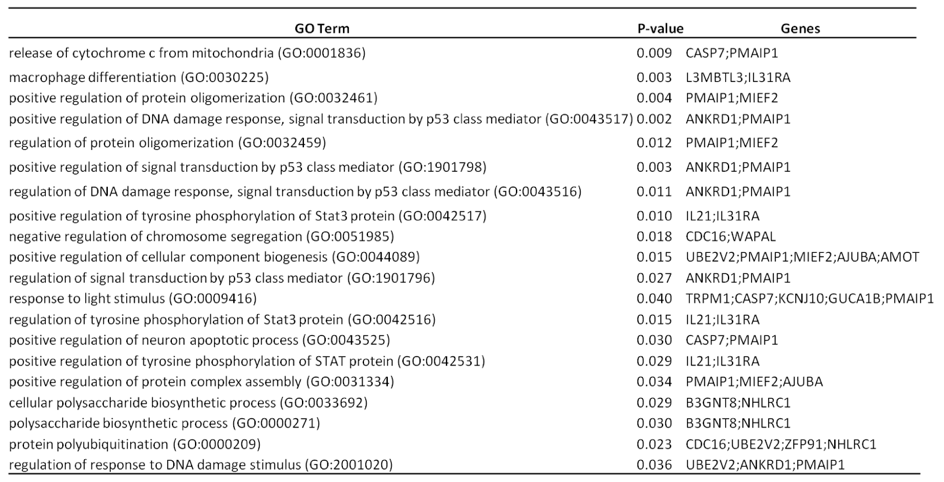
*

*Cluster 6*

*
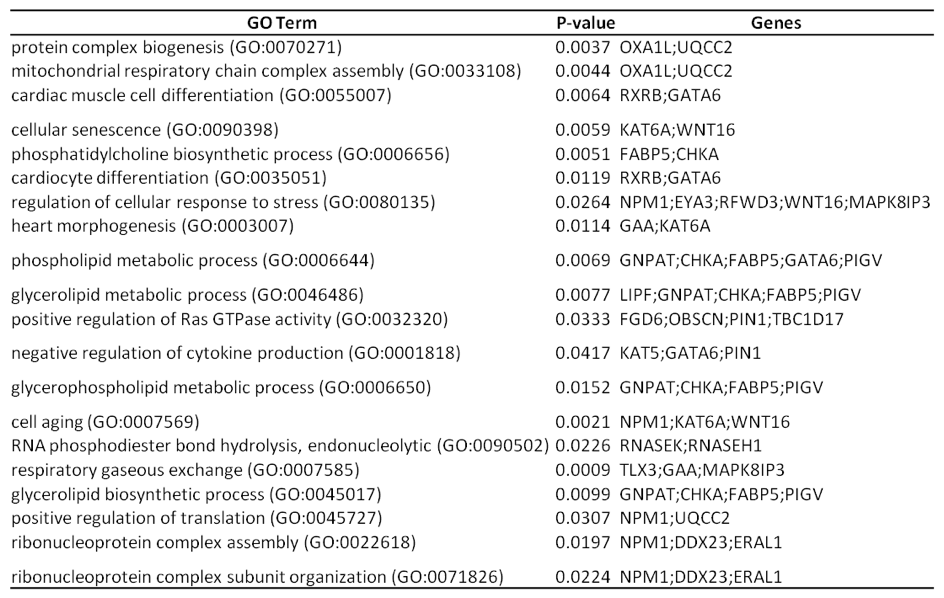
*

**Supplementary Table 2:** *Top Gene ontology terms from the gene clusters 1, 4 and 6 (Figure 9)*
